# CRTC2 modulates hepatic SREBP1c cleavage by controlling *Insig2a* expression during fasting

**DOI:** 10.1007/s13238-018-0538-3

**Published:** 2018-04-20

**Authors:** Yuanyuan Zhang, Yi Liu, Liqun Chen, Yiguo Wang, Jinbo Han

**Affiliations:** 1 MOE Key Laboratory of Bioinformatics, Tsinghua-Peking Center for Life Sciences, School of Life Sciences, Tsinghua University, Beijing, 100084 China; 2 School of Pharmaceutical Sciences, Tsinghua University, Beijing, 100084 China

**Dear Editor,**

Dysregulation of hepatic lipid and glucose production results in obesity, diabetes and nonalcoholic fatty liver disease. *De novo* lipogenesis and gluconeogenesis in the liver contribute, at least in part, to the dynamic homeostasis of lipid and glucose levels (Rui, 2014; Han and Wang, 2017). These processes are regulated at the transcriptional level by different transcription factors in response to various environmental cues, including hormones, nutrition and stress. Regulators of lipogenesis include the sterol regulatory element-binding proteins (SREBPs), which are members of the basic helix-loop-helix leucine zipper transcription factor family (Goldstein et al., 2006). There are three closely related SREBPs, SREBP1a, SREBP1c and SREBP2, among which SREBP1c and SREBP2 are the major isoforms expressed in the liver. SREBP1c mainly regulates fatty acid synthesis, while SREBP2 controls cholesterol production (Goldstein et al., 2006). SREBPs are synthesized as inactive precursors bound to the endoplasmic reticulum (ER), where they associate with the sterol cleavage activating protein (SCAP) (Goldstein et al., 2006). Upon sensing sterol demand, the SREBP/SCAP complex buds from the ER in a COPII-dependent manner and is transported to the Golgi, where SREBP processing occurs. The mature SREBP then translocates to the nucleus and activates the transcription of lipid biosynthesis genes. In the presence of excess sterol levels, the SREBP/SCAP complex binds to insulin-induced gene (INSIG) and is restricted to the ER (Goldstein et al., 2006). Thus, SREBP processing, shuttling of SREBP to the nucleus and transcriptional control of genes involved in cholesterol and fatty acid synthesis are tightly regulated in response to nutrition conditions.

SREBP1c processing is regulated not only by nutrient status but also by hormonal signaling during feeding and fasting (Horton et al., 1998; Li et al., 2010). These features distinguish it from SREBP2, processing of which is regulated by sterol levels. During feeding, insulin signaling promotes SREBP1c activity through two parallel pathways, one dependent on mTOR and the other dependent on *Insig2a* (Yecies et al., 2011; Han and Wang, 2017). The *Insig2* gene produces two alternatively spliced transcripts, *Insig2a* and *Insig2b*, which have different 5′-UTRs but share the same coding region (Yabe et al., 2003). Previous studies have shown that expression of *Insig2a*, the predominant *Insig2* isoform in liver, is enhanced during fasting and attenuated during feeding (Yabe et al., 2003). Furthermore, insulin signaling decreases *Insig2a* expression with concomitant enhancement of SREBP1c activity (Yabe et al., 2003; Yellaturu et al., 2009). Together, these results suggest that *Insig2a* is a potent regulator of SREBP1c activity during both feeding and fasting in the liver. Although both fasting and/or glucagon signaling can attenuate SREBP1c activity (Horton et al., 1998; Yabe et al., 2003), the molecular mechanism linking fasting to *Insig2a* expression is unclear.

In contrast to SREBP1c-mediated hepatic lipogenesis, gluconeogenesis is activated during fasting and shut down during feeding. Gluconeogenesis is partially regulated by cAMP response element-binding protein (CREB) and CREB regulated transcription coactivators (CRTCs) (Altarejos and Montminy, 2011). CRTC2, which is highly expressed in the liver, is dephosphorylated and shuttled to the nucleus during fasting, where it binds to CREB and induces gluconeogenic gene expression (Altarejos and Montminy, 2011). During feeding, CRTC2 is phosphorylated and sequestered in the cytoplasm through phosphorylation-dependent interactions with 14-3-3 proteins (Altarejos and Montminy, 2011). Our previous results showed that CRTC2 mediates mTOR-controlled SREBP1c processing and lipogenesis during feeding (Han et al., 2015). However, it is unclear whether CRTC2 affects SREBP1c processing during fasting.

To investigate the effect of CRTC2 on SREBP1c processing during fasting, we fasted mice for 12 h and evaluated SREBP1c processing. We found that higher levels of mature SREBP1c accumulate in *Crtc2*^−/−^ mice than in *Crtc2*^*+*/+^ mice following fasting (Fig. 1A). Expression of two SREBP1c-targeted lipogenic genes (*Fasn*, *Scd1*) was also elevated in *Crtc2*^−/−^ mice as judged by real-time quantitative PCR (qPCR) (Fig. 1B). In contrast to SREBP1c, levels of SCAP are comparable in *Crtc2*^+/+^ and *Crtc2*^−/−^ mice under fasted conditions (Fig. 1A). In addition to SCAP, INSIG family proteins, including INSIG1 and INSIG2, also play an important role in controlling SREBP1c processing (Yabe et al., 2002; Yabe et al., 2003). Therefore, we evaluated the hepatic expression of *Insig1*, *Insig2a* and *Insig2b* in *Crtc2*^−/−^ mice. *Insig2a*, but not *Insig1* or *Insig2b*, was dramatically decreased during fasting in *Crtc2*^−/−^ mice, which is similar to attenuated expression of gluconeogenic genes (*Pck1* and *G6pc*) (Figs. 1B and S1). In addition, fasting-induced effect on SREBP1c processing and *Insig2a* expression was blocked by H89, a PKA inhibitor (Fig. S2). Together, these results indicate that CRTC2 inhibits SREBP1c activity during fasting.

**Figure 1:**
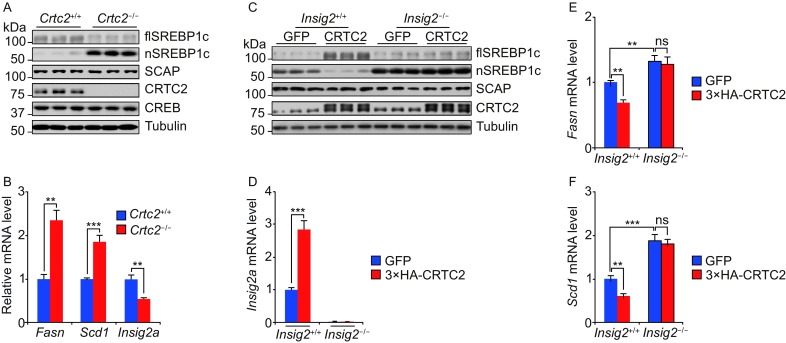
**CRTC2 inhibits SREBP1c processing via INSIG2 during fasting**. (A and B) Immunoblots showing hepatic amounts of full-length, inactive SREBP1c (flSREBP1c) and cleaved, active SREBP1c (nSREBP1c) in liver extracts (A) and qPCR results showing expression of lipogenic genes (*Fasn*, *Scd1* and *Insig2a*) (B) in 12 h fasted *Crtc2*^+/+^ and *Crtc2*^−/−^ mice. (C–F) Effect of 3×HA-CRTC2 overexpression on SREBP1c maturation (C), *Insig2a* expression (D), *Fasn* expression (E) and *Scd1* expression (F) in 12 h fasted *Insig2*^+/+^ and *Insig2*^−/−^ mice. ns, no significant statistical difference. Data are shown as mean ± s.e.m. **P < 0.01, ***P < 0.001, *n* = 8

We next asked whether CRTC2 modulates SREBP1c processing via INSIG2. Adenoviral expression of HA-tagged CRTC2 (3× HA-CRTC2) enhanced *Insig2a* expression and thus attenuated SREBP1c processing in wild-type mice during fasting (Fig. 1C–F). The inhibitory effect of CRTC2 on SREBP1c processing was abolished in *Insig2*^−/−^ mice (Fig. 1C–F). These results demonstrate that CRTC2 regulates SREBP1c processing in an INSIG2-dependent manner during fasting.

Considering that *Insig2a* is a predominant isoform of *Insig2* in the liver (Yabe et al., 2003) and CRTC2-dependent transcriptional effect on *Insig2a* expression, we tested whether the CRTC2/CREB complex directly regulates *Insig2a* expression. Overexpression of wild-type (WT) and constitutively active (CRTC2/AA) (Han et al., 2015) CRTC2 in the liver increased *Insig2a* expression; knockdown of *Crtc2* by short hairpin RNA decreased its expression (Fig. 2A and 2B), which is consistent with the results from *Crtc2* knockout mice (Fig. 1B). In addition, overexpression of ACREB, a dominant-negative form of CREB (Herzig et al., 2003), decreased *Insig2a* expression (Fig. 2C). Together, these results suggest that the CRTC2/CREB complex, as a whole, controls *Insig2a* expression.

**Figure 2:**
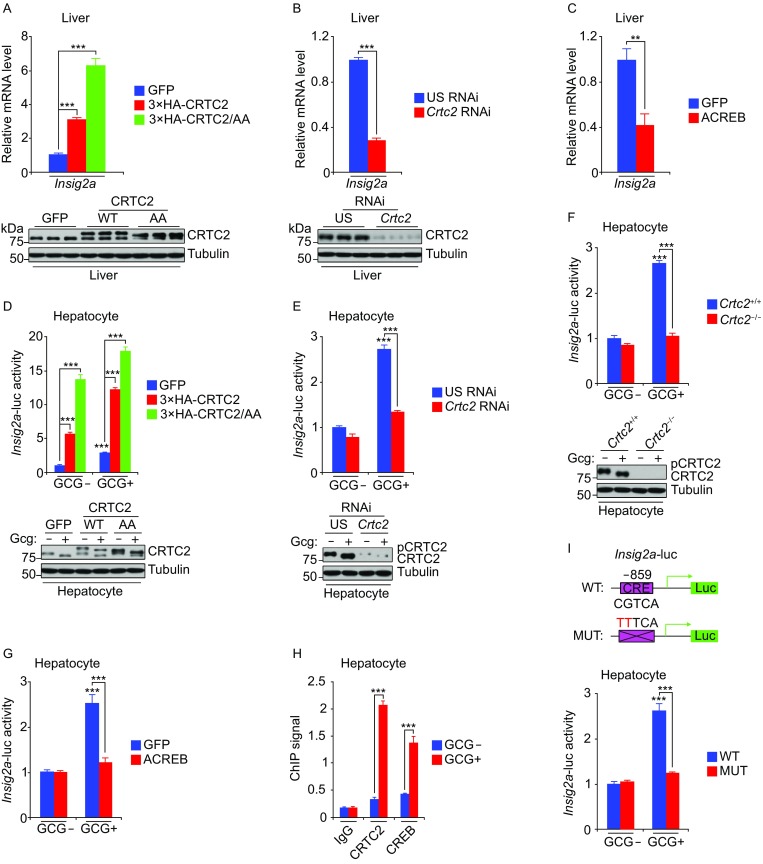
***Insig2a*** **is transcriptionally regulated by the CRTC2/CREB complex**. (A–C) Effect of wild-type (WT) 3×HA-CRTC2 and its nuclear-localized mutant (FLAG-CRTC2/AA, CRTC2 with double Ala mutations at Ser171 and Ser275) (A), *Crtc2* knockdown (B) or ACREB (C) on *Insig2a* expression in 12 h fasted mice. Bottom panels: Immunoblots showing the relative CRTC2 level. (D–G), Effect of 3×HA-CRTC2 and 3×HA-CRTC2/AA (D), CRTC2 deficiency by knockdown (E) or knockout (F), and ACREB (G) on *Insig2a*-luc activity in mouse primary hepatocytes. Bottom panels: Immunoblots showing the relative CRTC2 level. (H) Chromatin immunoprecipitation showing the occupancy of CRTC2 and CREB on the *Insig2a* promoter. (I) Top panel: Location of the wild-type (WT) CRE half-site and its mutant (MUT) within the mouse *Insig2a* promoter. Bottom panel: Relative luciferase activity with the wild-type or mutant *Insig2a* promoters in the presence or absence of glucagon. GCG, glucagon. US, unspecific. Data are shown as mean ± s.e.m. **P < 0.01, ***P < 0.001, *n* = 8

To further demonstrate whether the CRTC2/CREB complex autonomously regulates *Insig2a*, we used reporter assays in mouse primary hepatocytes. Overexpression of CRTC2 and CRTC2/AA enhanced *Insig2a*-luc activity following glucagon (Gcg) treatment, while *Crtc2* deficiency by knockdown or knockout attenuated *Insig2a*-luc activity (Fig. 2D–F). As shown in Figure 2G, overexpression of ACREB decreased *Insig2a*-luc activity in hepatocytes, consistent with the results in liver (Fig. 2C). These results show that the CRTC2/CREB complex autonomously controls *Insig2a* expression.

Having obtained evidence for a regulatory effect of the CRTC2/CREB complex on *Insig2a*, we performed chromatin immunoprecipitation (ChIP) studies to determine whether *Insig2a* is a direct target of CRTC2/CREB. Supporting this notion, glucagon stimulation dramatically increased both CRTC2 and CREB occupancy on the *Insig2a* promoter (Fig. 2H). CREB regulates gene expression by binding to palindromic (TGACGTCA) or to half-site (TGACG/CGTCA) cAMP-responsive elements (CREs) positioned within 1 kb of the transcriptional start site (Altarejos and Montminy, 2011). When we mutated a CRE half-site located at position − 859 of the *Insig2a* promoter, the *Insig2a*-luc reporter lost its ability to respond to glucagon (Fig. 2I), indicating that this element is critical for glucagon-induced transcriptional activation of *Insig2a*. Taken together, these results suggest that fasting activates CRTC2/CREB-controlled *Insig2a* expression, thereby attenuating SREBP1c processing.

It is well known that fasting or glucagon attenuates SREBP1c processing (Horton et al., 1998; Shimomura et al., 2000; Yabe et al., 2003), but the mechanism is unclear. Our results showed that the CRTC2/CREB complex controls *Insig2a* expression and thus attenuates SREBP1c processing during fasting (Fig. S3). This highlights the important role of the CRTC2/CREB complex in SREBP1c maturation during fasting. Meanwhile, our results fill the gap in our understanding of how glucagon or fasting signaling modulates SREBP1c processing.

## Footnotes

We thank Drs. Marc Montminy and Wei-min Tong for providing reagents. This work was supported by the National Natural Science Foundation of China (Grant No. 31500956).

All institutional and national guidelines for the care and use of laboratory animals were followed. Yuanyuan Zhang, Yi Liu, Liqun Chen, Yiguo Wang and Jinbo Han declare that they have no conflict of interest.

Yuanyuan Zhang and Jinbo Han designed the study and analyzed the data. Yuanyuan Zhang, Yi Liu and Liqun Chen performed the experiments. Jinbo Han wrote the paper. All authors reviewed and commented on the manuscript.

## Electronic supplementary material

Below is the link to the electronic supplementary material.

Supplementary material 1 (PDF 807 kb)

## References

[CR1] Altarejos JY, Montminy M (2011). CREB and the CRTC co-activators: sensors for hormonal and metabolic signals. Nat Rev Mol Cell Biol.

[CR2] Chen L, Wang K, Long A, Jia L, Zhang Y, Deng H, Li Y, Han J, Wang Y (2017). Fasting-induced hormonal regulation of lysosomal function. Cell Res.

[CR3] Goldstein JL, DeBose-Boyd RA, Brown MS (2006). Protein sensors for membrane sterols. Cell.

[CR4] Han J, Wang Y (2017). mTORC1 signaling in hepatic lipid metabolism. Protein Cell.

[CR5] Han J, Li E, Chen L, Zhang Y, Wei F, Liu J, Deng H, Wang Y (2015). The CREB coactivator CRTC2 controls hepatic lipid metabolism by regulating SREBP1. Nature.

[CR6] Herzig S, Hedrick S, Morantte I, Koo SH, Galimi F, Montminy M (2003). CREB controls hepatic lipid metabolism through nuclear hormone receptor PPAR-gamma. Nature.

[CR7] Horton JD, Bashmakov Y, Shimomura I, Shimano H (1998). Regulation of sterol regulatory element binding proteins in livers of fasted and refed mice. Proc Natl Acad Sci USA.

[CR8] Li S, Brown MS, Goldstein JL (2010). Bifurcation of insulin signaling pathway in rat liver: mTORC1 required for stimulation of lipogenesis, but not inhibition of gluconeogenesis. Proc Natl Acad Sci USA.

[CR9] Rui L (2014). Energy metabolism in the liver. Compr Physiol.

[CR10] Shimomura I, Matsuda M, Hammer RE, Bashmakov Y, Brown MS, Goldstein JL (2000). Decreased IRS-2 and increased SREBP-1c lead to mixed insulin resistance and sensitivity in livers of lipodystrophic and ob/ob mice. Mol Cell.

[CR11] Wang Y, Inoue H, Ravnskjaer K, Viste K, Miller N, Liu Y, Hedrick S, Vera L, Montminy M (2010). Targeted disruption of the CREB coactivator Crtc2 increases insulin sensitivity. Proc Natl Acad Sci USA.

[CR12] Yabe D, Brown MS, Goldstein JL (2002). Insig-2, a second endoplasmic reticulum protein that binds SCAP and blocks export of sterol regulatory element-binding proteins. Proc Natl Acad Sci USA.

[CR13] Yabe D, Komuro R, Liang G, Goldstein JL, Brown MS (2003). Liver-specific mRNA for Insig-2 down-regulated by insulin: implications for fatty acid synthesis. Proc Natl Acad Sci USA.

[CR14] Yecies JL, Zhang HH, Menon S, Liu S, Yecies D, Lipovsky AI, Gorgun C, Kwiatkowski DJ, Hotamisligil GS, Lee CH (2011). Akt stimulates hepatic SREBP1c and lipogenesis through parallel mTORC1-dependent and independent pathways. Cell Metab.

[CR15] Yellaturu CR, Deng X, Park EA, Raghow R, Elam MB (2009). Insulin enhances the biogenesis of nuclear sterol regulatory element-binding protein (SREBP)-1c by posttranscriptional down-regulation of Insig-2A and its dissociation from SREBP cleavage-activating protein (SCAP).SREBP-1c complex. J Biol Chem.

