# Supplementary material for: CRTC2 modulates hepatic SREBP1c cleavage by controlling Insig2a expression during fasting
Source: Protein Cell. 2018 Apr 20;9(8):729–32. doi: 10.1007/s13238-018-0538-3 (PMC6053347; doi:10.1007/s13238-018-0538-3)

MATERIALS AND METHODS

Mice

Mice were housed in colony cages with a 12 hr light/dark cycle in a temperature-controlled environment. For high-fat diet feeding experiments, regular diet (Research Diets, D12450B) was replaced with a diet containing 60 kcal% fat (Research Diets, D12492). *Crtc2*$^{-/-}$ mice have been described previously (Wang et al., 2010; Han et al., 2015). *Insig2*$^{-/-}$ (005939) and B6.Cg-*lep*^{ob}/J (000632) mice were purchased from Jackson Labs (Bar Harbor, ME). All mice were confirmed by PCR-based genotyping. All mouse experiments were approved by the Animal Care and Use Committee at Tsinghua University.

Plasmids and adenoviruses

INSIG2 was cloned from mouse liver cDNA. Adenoviruses (5 × 10^8 plaque forming units (pfu) GFP, 3×HA-CRTC2, 3×HA-CRTC2/AA (S171A, S275A), ACREB, *Crtc2* RNAi, or unspecific RNAi) (Herzig et al., 2003; Han et al., 2015) were delivered to 10-12 week old male C57BL/6J, *Crtc2*$^{+/+}$, *Crtc2*$^{-/-}$, *Insig2*$^{+/+}$ or *Insig2*$^{-/-}$ mice by tail vein injection. Mice were injected with adenovirus on day 0 and sacrificed on day 7. Mouse *Insig2a* promoter (from -972 bp to +37 bp) was cloned to produce *Insig2a*-luc. Mutagenesis was performed using a site-directed mutagenesis kit (Agilent Technologies). All constructs used in this study were confirmed by sequencing.

Quantitative PCR

Total RNA from mouse liver was extracted using RNeasy kits (Qiagen). cDNA was obtained using an iScript™ cDNA Synthesis Kit (Bio-Rad). RNA levels were measured with a LightCycler 480 II (Roche) as previously described (Han et al., 2015; Chen et al., 2017). The following primers were used for qPCR:

Actin-Forward: GTCCACCCCGGGGAAGGTGA
Actin-Reverse: AGGCCTCAGACCTGGGCCATT
Fasn-Forward: TGGTGGTGTGGACATGGTCACAGA
Fasn-Reverse: CCGAAGCTGGGGGTCCATTGTGTG
G6pc-Forward: GTGAATTACCAAGACTCCCAGGACTG
G6pc-Reverse: GATGGAACCAGATGGGAAAGAGGAC
Insig1-Forward: TCACAGTGACTGAGCTTCAGCA
Insig1-Reverse: TCATCTTCATCACACCCAGGAC
Insig2a-Forward: CCCTCAATGAATGTACTGAAGGATT
Insig2a-Reverse: TGTGAAGTGAAGCAGACCAATGT
Insig2b-Forward: CCGGGCAGAGCTCAGGAT
Insig2b-Reverse: GAAGCAGACCAATGTTTCAATGG
Pck1-Forward: ATGTGGCCAGGATCGAAAGCAAGAC
Pck1-Reverse: CTTTCATGCACCCTGGGAACCTGG
Scd1-Forward: CTGTACGGGATCATACTGGTTCCC
Scd1-Reverse: CAGCCGAGCCTTGTAAGTTCTGTG

Cell culture, luciferase assay and immunoblot

Mouse primary hepatocytes were isolated and cultured as previously described (Han et al., 2015; Chen et al., 2017). For reporter studies, Ad-*Insig2a*-luc infected hepatocytes (1 pfu per cell) were exposed to glucagon (100 nM) for 4 hrs. Immunoblotting was performed as described (Han et al., 2015; Chen et al., 2017). CRTC2, CREB, SREBP1, SCAP and Tubulin antibodies were as previously described (Han et al., 2015).

Chromatin immunopreciptation

Assays were performed as previously described (Chen et al., 2017). In brief, mouse primary hepatocytes were treated with glucagon (100 nM) or not for 90 min. Cells were cross-linked on the plates with 0.75% formaldehyde and chromatin was prepared essentially as described (Chen et al., 2017). For CREB and CRTC2 immunoprecipitation, rabbit polyclonal antibody was used along with rabbit IgG for negative controls. After removing crosslinks, DNA was extracted using phenol-chloroform, and CREB-target promoters were quantified using qPCR. The following primers were used: ChIP-forward: CCACTATGCATCATGTGGCTTTCTTA; ChIP-Reverse: TCTGATCTTGCCTTATTGGATATGCC. All signals were normalized to input chromatin signals.

Statistical analyses

Age- and weight-matched male mice were randomly assigned to different experimental groups. The number of animals used in each experiment is outlined in the corresponding figure legends. No animals were excluded from statistical analyses, and the investigators were not blinded in the studies. All studies were performed on at least three independent occasions. Results are reported as mean ± s.e.m. Comparison of different groups was carried out using the two-tailed unpaired Student's *t*-test. Differences were considered statistically significant at $P < 0.05$.

SUPPLEMENTARY INFORMATION

Figure S1: Effect of *Crtc2* deficiency on expression of lipogenic and gluconeogenic genes

(A-D), qPCR results showing expression of *Insig1* (A), *Insig2b* (B), *Pck1* (C) and *G6pc* (D) in 12 hr fasted *Crtc2*$^{+/+}$ and *Crtc2*$^{-/-}$ mice. Data are shown as mean ± s.e.m. $^{**}P < 0.01$, n = 8. *NS*, no significant statistical difference.

Figure S2: Effect of H89 on SREBP1c processing.

(A-C) Effect of H89 administration on SREBP1c processing (A) and gene expression of *Insig2a* (B) and *G6pc* (C) in liver extracts from mice. Mice were intraperitoneally injected with H89 (20 mg kg^{-1}) after 4 hr of fasting and further fasted for 8 hr before livers were harvested.

(D-E) Effect of H89 treatment on GCG-stimulated expression of *Insig2a* (D) and *G6pc* (E) in mouse primary hepatocytes. GCG: glucagon. Mouse primary hepatocytes were pretreated with 10 μM H89 for 30 min and incubated with 100 nM GCG for 4 hrs.

Data are shown as mean ± s.e.m. $^{*}P < 0.05$, $^{**}P < 0.01$, $^{***}P < 0.001$. n = 6.

Figure S3: CRTC2 regulates SREBP1c maturation through transcriptional control of *Insig2a*.

During fasting, the fully active CRTC2/CREB complex upregulates *Insig2a* expression and thus attenuates SREBP1c maturation.

Figure S1

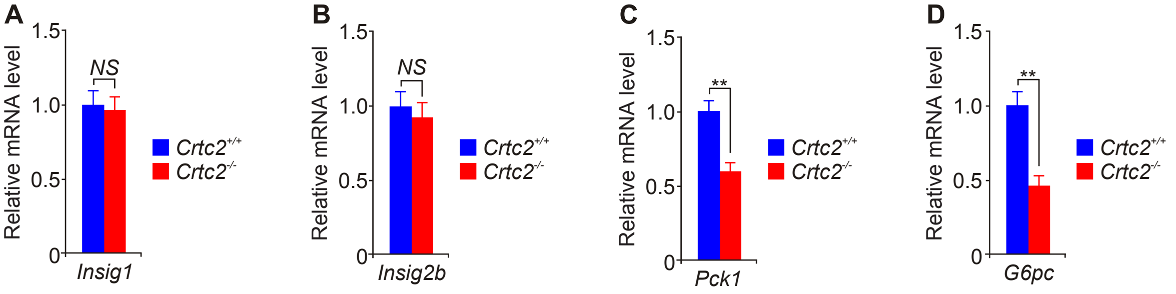

Figure S2

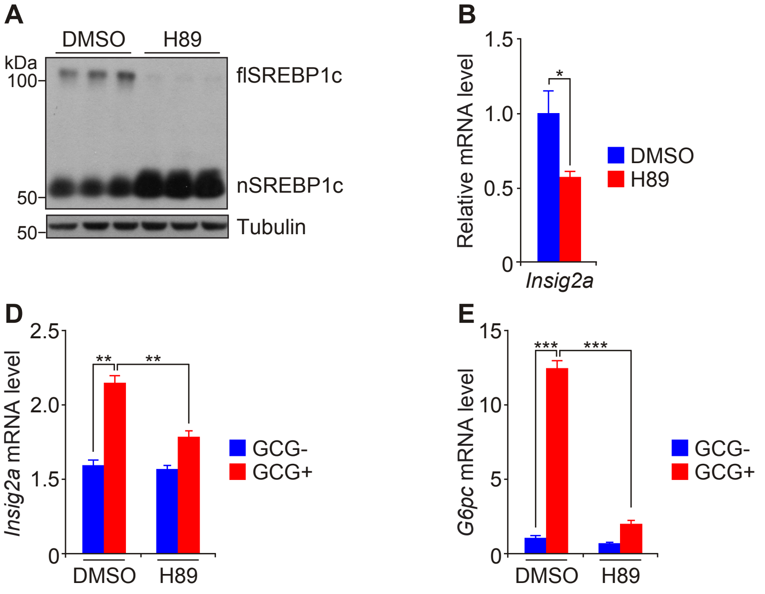

Figure S3

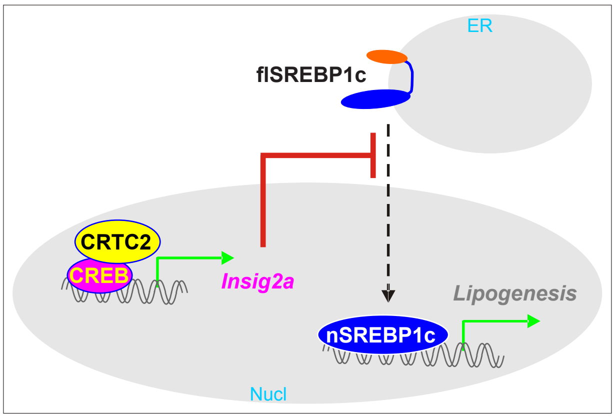

Supplement: Supplementary file 1 — Supplementary material 1 (PDF 807 kb) [file 13238_2018_538_MOESM1_ESM.pdf]
